# Challenges in diagnostic and catheter ablation of long RP supraventricular tachycardia with eccentric activation and decremental properties: a case report

**DOI:** 10.1186/s13256-025-05557-9

**Published:** 2025-10-21

**Authors:** Raymond Pranata, William Kamarullah, Giky Karwiky, Chaerul Achmad, Mohammad Iqbal

**Affiliations:** Department of Cardiology and Vascular Medicine, Faculty of Medicine, Hasan Sadikin General Hospital, Universitas Padjadjaran, Bandung, Indonesia

**Keywords:** Atrioventricular nodal reentrant tachycardia, Permanent junctional reciprocating tachycardia, Bystander accessory pathway, Supraventricular tachycardia, Long RP tachycardia

## Abstract

**Background:**

Long RP supraventricular tachycardia poses a significant diagnostic challenge because of overlapping electrophysiological features among differential diagnoses. Detailed evaluation with an electrophysiological study is essential for accurate diagnosis and effective management, particularly when initial ablation attempts fail to eliminate inducibility.

**Case presentation:**

A 40-year-old Southeast Asian male with a 5-year history of recurrent palpitations was referred for evaluation. Baseline echocardiography was normal. During symptomatic episodes, electrocardiography demonstrated long RP tachycardia. Electrophysiology study revealed eccentric atrial activation with decremental conduction, with the earliest A recorded at DD 9–10 (coronary sinus ostium/left posteroseptal region). Tachycardia cycle length was 410 ms, with a VA interval of 215 ms, AH interval of 93 ms, HA interval of 332 ms (AH/HA < 1), a VAV response during ventricular entrainment, PPI–TCL of 225 ms, and SA–VA of 194 ms. Ventricular reset did not terminate the arrhythmia and showed no atrial delay or advancement. Ablation at the coronary sinus ostium terminated the tachycardia but did not prevent reinduction. A subsequent slow pathway ablation was performed, during which slow junctional rhythm was observed. Post-ablation testing demonstrated crossover at 320 ms, while supraventricular tachycardia remained easily inducible with atrial S1 pacing at 400 ms. Given persistent inducibility, medical therapy was optimized and the patient was scheduled for advanced three-dimensional mapping and ablation. The leading differential diagnoses were atypical atrioventricular nodal reentrant tachycardia (fast–slow variant) with a bystander accessory pathway and permanent junctional reciprocating tachycardia with coexisting dual AV nodal physiology.

**Conclusion:**

This case illustrates the diagnostic complexity and management challenges of long RP supraventricular tachycardia, particularly in distinguishing atypical atrioventricular nodal reentrant tachycardia from permanent junctional reciprocating tachycardia. When initial ablation does not achieve full arrhythmia control, a stepwise strategy involving detailed electrophysiological evaluation, cautious ablation, and advanced mapping may be required to guide definitive therapy.

## Background

The differential diagnosis for long RP supraventricular tachycardia (SVT) includes focal atrial tachycardia (AT), atrioventricular reentrant tachycardia (AVRT), and atypical atrioventricular nodal reentrant tachycardia (AVNRT) [1]. In most cases, an electrophysiological (EP) study is necessary to discern the exact etiology. Accurate diagnosis is crucial for determining the appropriate ablation strategy. Here, we present a challenging case of long RP supraventricular tachycardia with eccentric activation and decremental properties. The most likely differential diagnoses include atypical AVNRT with bystander accessory pathway (AP) and permanent reciprocating junctional tachycardia (PJRT).

## Case presentation

A 40-year-old Southeast Asian male presented with a 5-year history of recurrent palpitations, often accompanied by dizziness and occasional syncope. At the time of admission, he was asymptomatic, with no chest pain or dyspnea. He denied exertional dyspnea, paroxysmal nocturnal dyspnea, orthopnea, or peripheral edema. Physical examination was unremarkable.

**Fig. 1.**
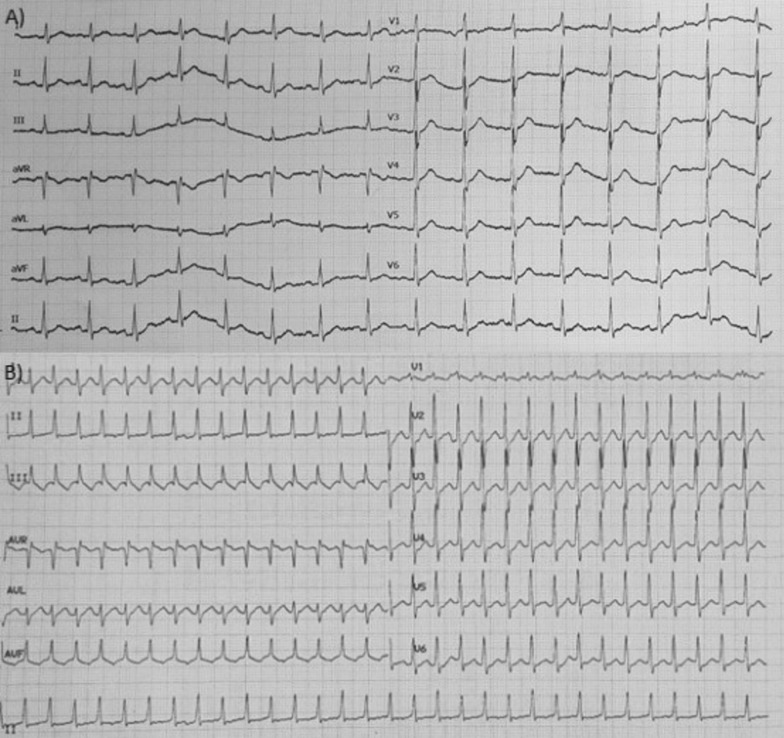
12 lead electrocardiogram. **A** during sinus rhythm, **B** during tachycardia

Echocardiography demonstrated normal chamber dimensions, preserved left ventricular (LV) systolic function (LVEF 58%), no regional wall motion abnormalities, and normal diastolic function. Valvular anatomy and function were normal. The mitral–tricuspid annular distance was within normal range, with low probability of pulmonary hypertension. Right ventricular (RV) systolic function was normal, and no features of Ebstein’s anomaly were noted. Electrocardiogram (ECG) in sinus rhythm showed an early precordial R-wave transition (Fig. 1A), while ECG during tachycardia demonstrated a long RP tachycardia with inverted P waves in leads III and aVF (Fig. 1B). Laboratory tests, including renal function, electrolytes, and thyroid-stimulating hormone, were all normal.

**Fig. 2:**
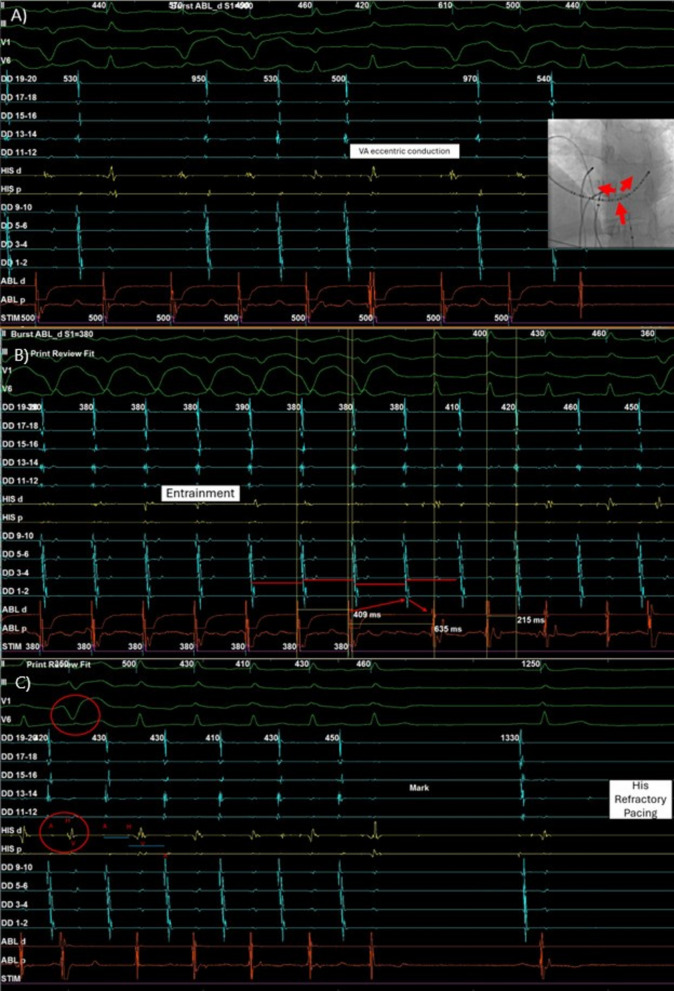
Intracardiac electrogram of electrophysiological study. **A** V pacing showed eccentric atrial activation and decremental properties with earliest A at DD 9–10, **B** Arrhythmia entrainment with ventriculo–atrial–ventricular response, **C** His refractory pacing

EP study revealed sinus rhythm at baseline with a cycle length of 849 ms. Ventricular pacing at S1 500 ms demonstrated eccentric atrial activation with decremental conduction, the earliest atrial activation at DD 9–10 (coronary sinus ostium/left posteroseptal region) (Fig. 2A). Atrial pacing at HRA S1 440 ms, HRA S1S2 500/360 ms, and ventricular pacing S1S1 600/480 ms induced SVT. The tachycardia cycle length was 410 ms, with a ventricular to atrial activation (VA) interval of 215 ms, atrial to His bundle electrogram (AH) interval of 93 ms, His bundle to atrial activation (HA) interval of 332 ms, and an AH/HA ratio < 1. Ventricular entrainment showed a ventriculo–atrial–ventricular (VAV) response, with a post-pacing interval minus tachycardia cycle length (PPI–TCL) of 225 ms and SA–VA of 194 ms (Fig. 2B). Ventricular reset did not terminate the tachycardia and produced no atrial advancement or delay (Fig. 2C). Atrial burst pacing yielded an atrial–His–atrial (AHA) response. No antegrade or retrograde jump preceded SVT induction. The tachycardia terminated spontaneously but was easily inducible during catheter manipulation throughout the EP study.

**Fig. 3:**
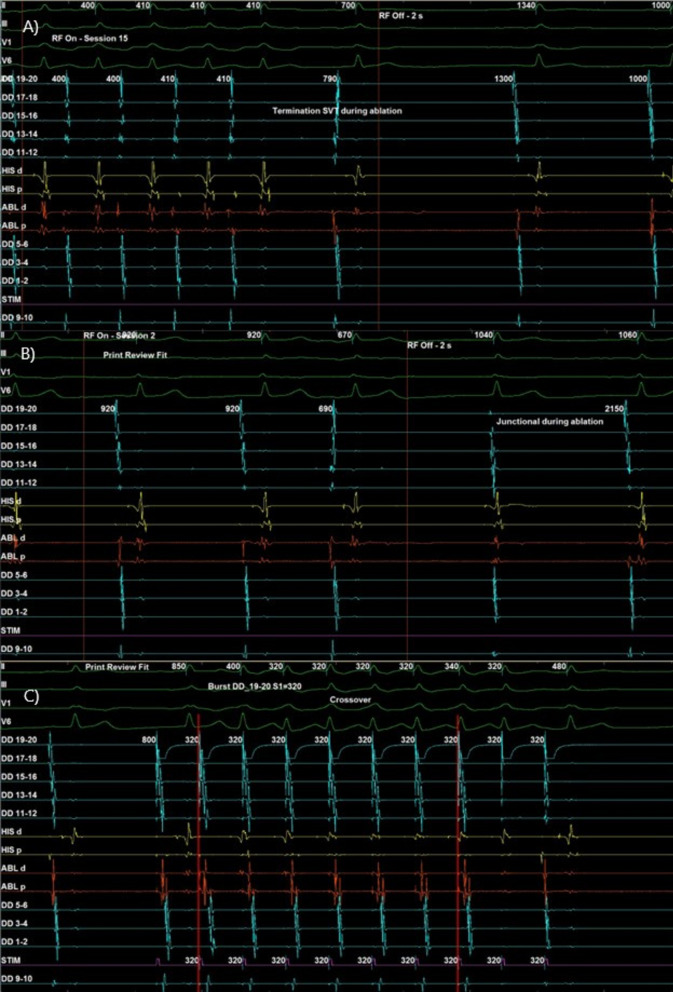
Catheter ablation. **A** Termination of supraventricular tachycardia during ablation at earliest A activation. **B** Slow junctional rhythm during ablation of slow accessory pathway. **C** Crossover phenomenon upon atrial burst remains observable despite ablation

Radiofrequency ablation was first attempted at the coronary sinus ostium, guided by the earliest A at DD9–10 during SVT. Tachycardia was terminated during ablation, but remained inducible (Fig. 3A). The ablation catheter could not be advanced further into the distal coronary sinus. Therefore, we decided to perform slow pathway ablation. A slow junctional rhythm was observed during ablation, confirming correct targeting (Fig. 3B). Postablation testing revealed crossover at 320 ms on HRA S1 pacing (Fig. 3C) and an AV block cycle length (AVBCL) of 300 ms. HRA S1S2 500/320 ms demonstrated the AV nodal effective refractory period (AVNERP). However, SVT remained easily inducible with atrial S1 pacing at 400 ms.

Given these findings, the decision was made to optimize medical therapy and defer further ablation. A 3D ablation procedure was planned if tachycardia persisted despite antiarrhythmic therapy. If performed, left atrial access would be obtained via transseptal puncture, and ablation would be guided by earliest activation mapping, with additional assessment for AV fusion in the event of an accessory pathway.

## Discussion and conclusion

This case presents a complex scenario of long RP SVT ablation. The main differentials include AVNRT fast-slow pathway with bystander AP and PJRT with bystander dual AV nodal physiology. Inducible SVT persisted after ablation at the site of earliest atrial activation near the coronary ostium and after successful slow pathway ablation. Considering the risks associated with left-sided ablation and the need for 3D mapping—which is not readily available at this hospital—we decided to plan an elective procedure if the tachycardia persists despite antiarrhythmic therapy.

Ventricular pacing with S1 500 ms showed eccentric atrial activation with decremental properties and earliest A at DD 9–10, suggesting an AP at the coronary ostium or left posteroseptal region. A VAV response supported AVRT or AVNRT and excluded atrial tachycardia (AT). The PPI–TCL was 225 ms, which is > 110 ms, indicating the mechanism was unlikely AVRT. However, in PJRT, decremental AP conduction may confound these findings. Nevertheless, Ho *et al*. reported that PPI–TCL was < 125 ms in five out of six patients with PJRT, whereas all patients with atypical AVNRT had PPI–TCL > 125 ms [2]. The VA interval was 215 ms; a VA > 60 ms favors fast–slow AVNRT or AVRT, and a VA > 200 ms suggests conduction via a slow pathway. The AH/HA ratio was < 1, consistent with fast–slow (atypical) conduction [3]. In addition, Ho *et al*. demonstrated that VA was < 85 ms in four of six patients with PJRT and in none of fourteen atypical patients with AVNRT [2]. The SA–VA interval was 194 ms, supporting AVNRT. Atrial burst pacing showed an AHA response, ruling out junctional tachycardia, which typically produces an AHHA pattern. His-refractory pacing and ventricular reset showed no atrial advancement or delay. Although advancement, delay, or termination would support AP involvement in AVRT or PJRT, absence of these findings does not exclude AP. Based on these findings (Table 1), AT and AVRT were excluded, while atypical AVNRT and PJRT remained possible.

**Table 1:** Differential diagnosis on the basis of electrophysiological study

AVNRT	AVRT	AT	PJRT
(+) VAV response	(+) VAV response	(−) VAV response	(+) VAV response
(+) PPI-TCL > 110 ms	(+) Eccentric activation	(±) Eccentric activation	(+) Eccentric activation
(+) Decremental properties	(+) VA interval > 60 ms	(−) Termination ends with V	(+) Decremental properties
(+) Ventricular reset → no atrial delay/advancement	(−) PPI-TCL > 110 ms	(−) No warm-up or cooldown	(?) Ventricular reset → no atrial delay/advancement
(+) SA-VA > 85 ms	(−) Ventricular reset → no atrial delay/advancement		(?) PPI-TCL > 110 ms [Decremental]
(−) No antegrade or retrograde jump during induction, no echo beat	(−) Decremental properties		(?) SA-VA > 85 ms [Decremental]
(−) Eccentric activation	(−) SA-VA > 85 ms		

*AT* atrial tachycardia, *AVNRT* atrioventricular nodal reentrant tachycardia, *AVRT* atrioventricular reentrant tachycardia, *PPI–TCL* post pacing interval–tachycardia cycle length, *PJRT* permanent reciprocating junctional tachycardia

Ablation guided by earliest A at DD 9–10 terminated SVT during energy delivery, but tachycardia remained inducible. Slow pathway ablation at the right inferior extension during sinus rhythm produced a slow junctional rhythm, indicating successful lesion placement. Postablation testing showed crossover (320 ms) and AVBCL (300 ms) on HRA S1 pacing, but SVT remained easily inducible with atrial S1 400 ms. Based on the available data, we were able to narrow the possibilities into (1) AVNRT fast-slow pathway with bystander AP, (2) PJRT with bystander dual AV nodal physiology, and (3) AVNRT fast-slow pathway (Table 2). The data suggest either a decremental AP or a leftward inferior extension slow pathway. Dual AV nodal physiology was demonstrated by crossover despite the absence of AH jump, and slow junctional rhythm during ablation confirmed successful targeting of the slow pathway. The atypical AVNRT may therefore involve a leftward inferior extension slow pathway, explaining the earliest A activation at DD 9–10 during ventricular pacing.

**Table 2:** Differential diagnosis on the basis of electrophysiological study and catheter ablation

AVNRT (fast-slow pathway) with bystander AP	PJRT with bystander dual AV nodal physiology	AVNRT fast-slow pathway
(+) Crossover [slow pathway]	(+) Incessant, easily induced	(+) Ventricular reset → no atrial delay/advancement/termination
(+) Slow junction rhythm during slow pathway ablation	(−) No tachycardia induced cardiomyopathy [Normal Echocardiography]	(+) Slow junction rhythm during slow pathway ablation
(?) Narrow QRS		(+) Crossover [slow pathway]
		(−) No AH critical time for SVT induction

*AP* accessory pathway, *AVNRT* atrioventricular nodal reentrant tachycardia, *PJRT* permanent reciprocating junctional tachycardia, *SVT* supraventricular tachycardia

Epidemiologic studies indicate that PJRT is more common in women, and incessant tachycardia may lead to tachycardia-induced cardiomyopathy[4]. In this case, echocardiography was normal, which does not exclude PJRT but makes it less likely. Taken together, the data suggest that the main differentials are AVNRT with fast–slow pathway and bystander AP, or PJRT with bystander dual AV nodal physiology. If subsequent ablation is to be performed, the left atrium should be accessed with transeptal puncture and AV fusion should be looked at in case of AP. The site for ablation will be based on earliest activation time and the AV fusion [5].

We present a complex case of long RP SVT ablation with the main differentials being AVNRT with fast–slow pathway and bystander AP, and PJRT with bystander dual AV nodal physiology. In such cases, left atrial access and 3D mapping based on earliest activation may be required for definitive treatment.

## Data Availability

Available upon reasonable request to the corresponding author.
